# Identification and Whole-Genome Sequencing of a Monkeypox Virus Strain Isolated in Israel

**DOI:** 10.1128/MRA.01524-19

**Published:** 2020-03-05

**Authors:** Inbar Cohen-Gihon, Ofir Israeli, Ohad Shifman, Noam Erez, Sharon Melamed, Nir Paran, Adi Beth-Din, Anat Zvi

**Affiliations:** a Department of Biochemistry and Molecular Genetics, Israel Institute for Biological Research, Ness Ziona, Israel; b Department of Infectious Diseases, Israel Institute for Biological Research, Ness Ziona, Israel; KU Leuven

## Abstract

We report the whole-genome sequence of a monkeypox virus strain isolated in Israel. The strain was isolated in 2018 from a patient travelling back from West Africa. The virus was fully sequenced on the Illumina MiSeq and Oxford Nanopore Technologies MinION platforms.

## ANNOUNCEMENT

Monkeypox virus (MPXV) is a member of the *Orthopoxvirus* genus of enveloped large viruses, harboring a large double-stranded DNA genome. Human monkeypox is a zoonotic disease manifested by disseminated pustular lesions, resembling smallpox but with significantly reduced morbidity and lower death rates (between 1% and 10%). MPXV is closely related to other human orthopoxviruses, such as variola, cowpox, and vaccinia viruses. MPXV isolates from the central African Congo basin are typically more virulent than isolates from western Africa (1). West Africa, and specifically Nigeria, is presently suffering an unusually large and lethal MPXV outbreak. Since the end of 2017, a total of 269 suspected cases, including 115 confirmed cases and 7 MPXV-associated deaths, have been reported (https://www.who.int/csr/don/05-october-2018-monkeypox-nigeria/en).

Here, we report the identification and whole-genome sequencing of an MPXV strain isolated in Israel. The subject had disposed of rodent carcasses at his residence in Port Harcourt, Nigeria. He returned to Israel after 1 week; a few days later, he was hospitalized with synchronic pox-like lesions, which appeared following two peaks of fever. Pustule swab samples were analyzed by PCR, according to the method described by Shchelkunov et al., and were positive for MPXV (West African clade) (2, 3).

A Nextera XT paired-end library (Illumina) was prepared using 1 ng of DNA extracted from the patient’s pustule swab, using a QIAamp DNA minikit (Qiagen). The library was sequenced on the MiSeq platform using paired-end sequencing, with a read length of 150 nucleotides (nt) and a mean insert size of 275 nt. This produced 2,684,322 reads, with 54.2× coverage in mapping to MPXV. FastQC (https://www.bioinformatics.babraham.ac.uk/projects/fastqc) with default settings was used for quality control of the data. Human reads were filtered out using Bowtie 2 (4) with default parameters. For Nanopore sequencing, libraries were prepared from 5 ng of genomic DNA (gDNA) using a rapid PCR barcoding kit (SQK-RPB004; Oxford Nanopore Technologies), without fragmentation, and were sequenced on a MinION device using an MK1 R9.4 flow cell, following the protocol for 1D gDNA, which produced 6,940 reads with a mean length of 2,964.3 nt (standard deviation, 1,503.9 nt) and a maximum read length of 9,647 nt. A hybrid Nanopore-Illumina *de novo* assembly was performed using SPAdes (5), with the following parameters: t 4, m 32, and k 31,51,71. The final genome assembly was done by manually inspecting and curating the sequences of the large inverted terminal repeats obtained by the *de novo* hybrid assembly and adding them to the central genome. The complete genome of the Israeli MPXV isolate consists of 197,417 bp (G+C content, 33.2%). The genomic sequence of this MPXV strain was compared to all other complete MPXV genomic sequences available in the NCBI database by using BLAST (6), which revealed that the sequence differs from that of the closest strain, Nigeria-SE-1971 (accession no. KJ642617), by 470 single-nucleotide polymorphisms (99.76% genome similarity).

Monitoring large viral outbreaks is a worldwide concern. The MPXV strain whose sequence was reported here is part of such an outbreak. The data presented here will enrich the existing data on MPXV sequences and improve our awareness regarding the dissemination of such an outbreak.

### Data availability.

The genome sequence of the Israeli MPXV isolate was submitted to NCBI GenBank and is available under accession no. MN648051. The raw reads were submitted to and are available in the NCBI Sequence Read Archive (accession no. PRJNA587334).
